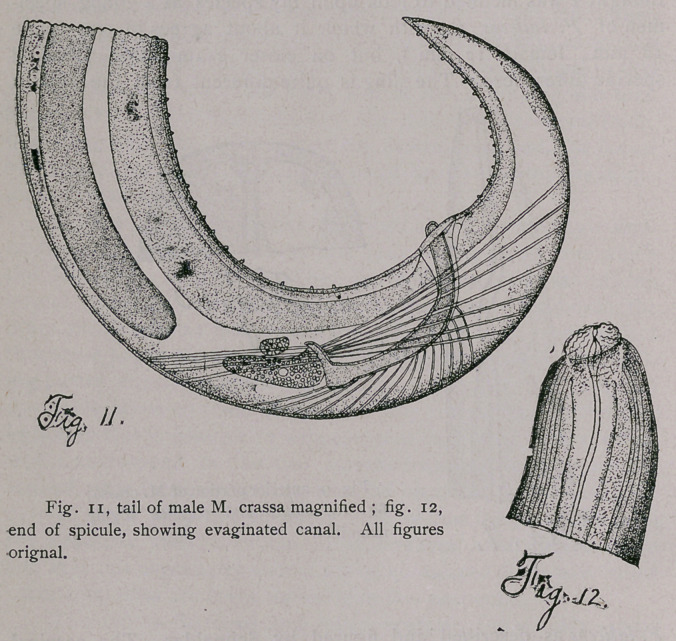# Notes on Parasites

**Published:** 1892-09

**Authors:** Charles W. Stiles

**Affiliations:** Washington, D. C.


### THE JOURNAL
OF
### COMPARATIVE MEDICINE AND VETERINARY ARCHIVES.

VOL. XIII. SEPTEMBER 1892. No. 9.

### NOTES ON PARASITES. No. II.

BY CHARLES W. STILES, PH. D.

A preliminary note on the following observations was read before the Société Zoologique de France, on June 9, 1891. A few days previous to presenting the paper (1) before the society I was summoned by cablegram to return to America, and was unable to prepare the illustrations in time for publication with the preliminary note, and since returning I have delayed the publication of my figures in the hope of obtaining more material. As this has not been possible, and as one of the parasites mentioned (*Coccidium bigeminum*) has since then appeared in print several times, although without figures, I have decided not to delay the publication any longer, but will now give the type figures of the parasites in question.

(*a*) *Coccidium bigeminum*, Stiles, '91. The twin-coccidia. (Syn. corpuscules geminés, Finck, 1854; cytospermium villosum intestinalis canis, Rivolta; coccidie geminé, Railliet et Lucet, 1890.)

In the literature on the sporozoa we find frequent mention of a parasitic protozoa in the intestinal tract of domestic dogs (R. Virchow, Rivolta, R. Leuckart, Railliet, etc.): R. Virchow (2) describes a case where the intestinal lining was fairly packed with coccidia; several authors mention the presence in the dog of *C. perforans*, the intestinal coccidium of rabbits; Rivolta named a *Cytospermium villosum intestinalis canis*, which Railliet and Lucet (3) consider identical with a parasite they found in the intestinal villosities of Parisian dogs. They recognized that it was a true coccidium, and also that it generally occurs in pairs, a fact which they were inclined to explain by assuming a division of the original cell. Shortly after R. and L. published their paper, I was working on the coccidia in Balbiani's laboratory, and Railliet very kindly gave me some of his original material for further study, in the course of which I came to the same results that Railliet found, and changing the French expression he used, when referring to the parasites, into Latin, I named the organisms *Coccidium bigeminum.* Since the appearance of my preliminary note, Railliet and Lucet (4) have published a second note on the subject, in which they state that this parasite was originally found in the villosities of cats by Finck in 1854, (5) who gave a very good description of it, but who, I may add, did not understand its true nature, for he called the parasites "*corpuscules geminés*," and doubting their parasitic nature, he assumed that they stood in some relation to the mechanism of fat

Fig. 1., a portion of a villus of a dog's intestine, showing a number of parasitic C. bigeminum; figs. 2–7, various stages in the development of C. bigeminum—for explanation see the text. All highly magnified and type figures.

absorption. Two figures of the bodies are also given, and there can be no doubt that they are identical with *C. bigeminum.* In the last paper by Railliet and Lucet, (4) and also in my review in the December number of this Journal (6), the suggestion is offered that the coccidia of the dogs' intestines referred to by various authors as *C. perforans* are probably identical with *C. bigeminum*, at least, Railliet, Lucet, and I have examined a large number of dogs, and have never found in them any species except *C. bigeminum.*

Last spring I examined ten dogs in Paris, and found the twin coccidia present in four of them. They are situated under the epithelial layer of the villosities.

Fig. 1 represents a portion of a villosity containing several parasites.

Fig. 2 shows a single Coccidum (0.015 mm.), evidently immediately before its division into the twin coccidia. A well defined membrane is present ; the contents of the cyst are granular.

Fig. 3 shows a parasite, after the granular mass was divided into two cells, each measuring 0.0079 by 0.0079.

In Fig. 4 each of the twins has formed about itself a membrane of its own, and the contents of one of the cysts have receded from the cyst wall preparatory to dividing into sporoblasts. Each twin then divides into four sporoblasts (Fig. 5, 6), and a protoplasmic rest (reliquat de segmentation). As will be seen by comparing the figures, the sporoblasts vary considerably, both in regard to size and relative position. In several cases I noticed that only one of the twins reached its full development, while the other degenerated (Fig. 7). This reminds one of the case described by Balbiani (7). In the intestinal epithelium of *Crytops punctatus*, Balbiani discovered a sporozoa in which there is a similar division of the original body, and in which only one of the resulting smaller bodies came to maturity, while the other aborted as in the case here described.

The measurements given by Railliet and Lucet vary from 0.012-0.015 mm. long by 0.007-0.009 mm. wide ; Finck's measurements are 0.008-0.01 mm. by 0.007-0.009 mm.

My own measurements in mm. are as follows :

| Single cysts. (evidently before division.) | Twin cysts. |
| --- | --- |
| 0.0135 by 0.0093 | 0.0106 by 0.0093 |
| 0.0139 by 0.0079 | 0.0106 by 0.0103 |
| 0.0139 by 0.0099 | |
| 0.0159 by 0.0086 | |

The variation in the first column must be explained, both by differences in the stage of development of the parasites examined and in individual variations, while the variation in the measurements of the twin cysts must be explained by individual variation alone, for the membranes were already formed, and the protoplasmic mass had receded, so that there was no chance for the parasites to increase in size by growth.

This parasite occurs in dogs (Railliet, Lucet, Stiles), in cats (Railliet, Lucet, Finck), and in *Putorius putorius*, the French "putois" (Railliet and Lucet), and Railliet and Lucet (4) admit three varieties which the parasite assumes, according to the host in which it is found, the three varieties differing only in point of size, the measurement being that of the entire double cyst :

| | | |
|---|---|---|
| Var. *canis* | 0.012-0.0159 mm. | by 0.007-0.0099 mm. |
| Var. *felis* | 0.008-0.010 " | by 0.007-0.009 |
| Var. *putorii* | 0.008-0.012 " | by 0.006-0.008 |

I have changed the measurements of Railliet and Lucet in the case of Var. *canis* by adding a 9 in the fourth decimal place, to agree with the largest measurement I found.

Balbiani and others have proven that the four sporoblasts in other coccidia, each divide further into two sickle-form spores. I was unable to find any stages of this division in the new species, but the division probably takes place after the coccidia leave the host.

I have examined a number of dogs in Washington, D. C., for these parasites, but as yet have not been able to find them.

When present in small numbers the parasites can, of course, have no appreciable effect upon the dog ; as to whether a heavy infection can do great injury has, as yet, not been demonstrated, but we can easily imagine that such an infection as Virchow describes in one place can work to the detriment of the host.

Grassi (8) has also described a parasite (*Coccidium Rivoltæ*) from the cat, which Thélohan (9) does not consider a Coccidium, but a *Cyclospora* (two sporoblasts with four falciform bodies).

Type material deposited in the collection of the Bureau of Animal Industry, in the U. S. National Museum, and in my private collection.

(*b*) *Dispharagus* (*Filaria*) *gasterostei*, Stiles, 1891.

While pursuing helminthological studies under Geheimrath Rudolph Leuckart, in Leipzig several years ago, I found a few specimens of a Filaria (Dispharagus) encysted and free in the body cavity of the stickle-back (Gasterosteus aculeatus) of the Baltic Sea. In transporting my material to America an accident happened to it by which I lost all but two specimens, one male in good preservation, and one female much distorted.

The parasite belongs to that group of the Filaridæ which possesses a sling (Krause) on its anterior portion, and to Dujardin's genus *Dispharagus*, if we wish to consider that as a good genus, a point upon which authors are divided.

The male is 10 mm. long and 0.24 mm. broad (middle of the body). The cuticle is finely annulated, each ring being 0.005 broad, and shows also several other differentiations.

Two extremely small lips bound the mouth laterally. A short distance back of the mouth are found four submedian papillæ (fig. 8).

A double sling (fig. 8) extends backward from the mouth on each side of the body, each sling describing a figure much like a vertical optical section, through an elongated gastrula. The slings are formed by two parallel bands ; the internal band is continuous, the external is interrupted in the lateral line, and is somewhat broader than the internal band. From the outer edge of the former extend longer and shorter parallel lines across the narrower band

FIG. 8.

Fig. 8, the sling on the head of D. gasterostei, showing the curvatures ; two of the sense papillae are also visible. Type figure.

in such a manner as to give the appearance of a lobed comb. The slings present four curvatures, two cephalad, two caudad (fig. 8). The cephalad curvatures are respectively 0.168 mm. and 0.33 mm. from the mouth ; the caudad curvatures are 0.275 mm. and 1 mm. from the mouth. Two hooks are found in the lateral lines, one on each side, directly back of the slings, 0.94 mm. from the oral extremity of the parasite. Each hook is two-pronged, measures 9.073 mm. long, 0.02 mm. broad (at the base), and projects through a circular opening in the cuticle.

The lateral lines are provided with wings which begin 1 mm.

from the mouth. At first very small, they enlarge rapidly to a maximum width, then decrease slowly in size, until finally about 7 mm. from the mouth they disappear entirely.

Two rows of finger-like projections of the hypodermis extend into the lateral folds, one dorsal, the other ventral of the lateral line. The ventral and dorsal projections sometimes correspond, but need not necessarily do so. The double projection thus formed has the appearance of a stripe placed transversely at right angles to the axis of the worm, and measures 0.0335 by 0.0067 mm. when viewed from the end, and attains a length of 0.079 mm., as seen in dorsal or ventral aspect of the worm. Each finger-like projection corresponds to a ring of the body, and in fact is nothing else than the extension of the hypodermis of the ring into the lateral folds. Beginning with the point where the lateral folds end, the dorsal and ventral halves of the rings dovetail into each other as in the case of other nematodes.

Besides the cervical hooks described above, I found two similar hooks in the caudal half of the body of the male, from which this description is written. They lie immediately dorsal of the lateral fold, one on the right side 4.5 mm. from the mouth, the other on the left, 0.7 mm. nearer the tail. It is a very curious fact that these hooks are asymmetrical. Furthermore, the corresponding points directly opposite the hooks on the left and right respectively, show no trace whatever of any rudimentary or broken hooks, so it is impossible to suppose that two other hooks, which would correspond to the two asymmetrically placed hooks described, were formerly present, but had been lost during the wanderings of the parasites, unless we suppose that the parasite has, in the meantime, shed its cuticle. It is, however, hardly necessary to add that this asymmetry should not be considered diagnostic unless other specimens are found agreeing in this particular.

The tail is flattened and rolled ventrally; side folds extend forwards for 0.42 mm. from the tip of the tail.

Instead of there being but one spicule present, as I wrote in my preliminary note, my original drawings and type specimen show that there are two spicula of unequal size; the longer measures about 0.7 mm. long by 0.012 thick, the shorter, 0.16 mm. long by 0.027 thick. These spicules emerge from the body 0.27 mm. from the tail.

Nine pairs of papillæ, four præ-anal, five post-anal, found on the flattened ventral side of the tail. The papillaæ vary somewhat in form.

In regard to the females I can simply state that they measured 12-16 mm. in length by 0.24 mm. in breadth. The cephalic slings are larger than in the male, as would be expected on account of the larger body of the female. Several specimens were found where the anterior portion was telescoped, giving a very curious and complicated appearance to the head.

Schneider (10) gives nine species of Filaria with cephalic slings, all of which are found in the œsophagi of birds. At first thought I was inclined to look upon my species as a young specimen of *F. laticeps R.*, with which it about agrees in size (male 10 mm., female 12 mm.), but on closer examination I found specific differences. The sling is quite different from the sling of

Fig. 9.

Fig. 10.

Fig. 9, anterior portion of M. crassa; fig. 10, mouth and papillae highly magnified.

*F. laticeps* as described and figured by Schneider. The cervical hooks are two-pronged, while Schneider figures those of *F. laticeps* as three-pronged. Von Linstow (11) has also described a very similar parasite, *Filaria involuta*, from the stomach of *Strix flammea*, an owl, but that, too, has a trifurcate papillæ (spine), and lacks the small caudad curvature in the sling on the head shown in the figure of *Dispharagus gasterostei*.

So far as I know this is the only Filaria (Dispharagus) with a cephalic sling which has been found in fish. The first thought which strikes one, is, as I intimated above, that the larval forms of this group will be found in fish. However, that is mere speculation at present.

The type material is in my private collection.

(*c*) *Mermis crassa* v. Linstow, 1889.

In one of Prof. Balbiani's aquaria containing a large number of larvæ of *Chironomus plumosus* L.,* I noted several specimens of a nematode, which were soon determined as *M. crassa* v. L. Examing the aquarium for several days in succession, and taking out

Fig. 11.

Fig. 11, tail of male M. crassa magnified; fig. 12, end of spicule, showing evaginated canal. All figures orignal.

Fig. 12.

each the worms which had escaped from the Diptera larvæ during the twenty-four hours previous, I was able to obtain quite a number of the specimens of the parasite.

This helminth was first described and named by von Linstow (12) in 1889, who found some of the free living specimens in the neighborhood of Göttingen. In a more recent article (13) v. Linstow mentions that he found the larvæ of this parasite in the body cavity of the larvæ of *Chironomus plumosus*. He was uncertain

* The small "red worm" fed to gold fish.

whether the worms escaped from their host during the larval stage of the latter or after the insect had reached its adult form, yet he inclined to the former opinion. v. Linstow states that Mermis larvæ have twice before been noticed in this same insect, in the adult insect by v. Siebold (14), and in the larvæ by Kraemer (15).

The measurements of the worm (all females) as given by v. Linstow vary from 5.5 mm. long by 0.15 broad (larva in insect larva) to 59 mm. by 0.9 mm. (free living mermis). Of the specimens I found, the males varied from 19 mm. to 25 mm. in length, the females from 23 mm. to 90 mm. in length.

As yet the males of *Mermis albicans*, *M. lacinulata*, and *M. paludicola* have not been seen. v. Linstow gives as characteristic of all these males, that two equally large spicula are present. In the males of *M. crassa*, however, I found only one spiculum present (Fig. 11). In several cases I distinguished a canal running through the spiculum and emptying, by means of an aperture, at the external tip. In three cases the canal was somewhat evaginated through the aperture (Fig. 12).

The tail of the male is curved ventrally and shows a large number of papillæ, which, however, are not constant in regard to their number or relative position. In general, however, they were arranged in a double middle row and two lateral rows back of the anus; in front of the anus these four rows were continued, and two more lateral rows were added.

WASHINGTON, D. C., Feb. 20, 1892.

LITERATURE.

1. *C. W. Stiles*, Note préliminaire sur quelques parasites (Bull d. l. Soc. Zool. de France, 1891, p. 163–165). Review by Braun, Centralblatt fur Bakteriologie und Parasitenkunde, 1891, X p. 392.

2. *R. Virchow*, Helminthologische Notizen—3. Ueber Trichina spiralis (Virchow's Archiv., 1860, XVIII, p. 330, see p. 342 and 527).

3. *A. Railliet* et *A. Lucet*, Observations sur quelques coccidies intestinalis. (Comp. rend. d. l. Soc. d. Biol., 1890, p. 660, 661).

4. *R. et L.*, Note sur quelques espèces de coccidies encore peu étudiées. (Bull. d. l. Soc. Zool. de France, 1891, p. 246–250).

5. *H. Finck*, Sur la physiologie de l'épithelium intestinal. Thèse de méd. Strasbourg (2) no. 324, 1854.

6. *C. W. Stiles*, I. Review of recent Publications in Medical Zoology. (This Journal, p. 692).

7. *Balbiani*, Journal de l'Anatomie et de la Physiologie de l'Homme et des Animaux, 1889, XXV, p. 42–44.

8. *Grassi*, Sur quelques protistes endo-parasites (Arch. Ital. de Biologie, 1882, p. 438).

9. *Thélohan*, Sur deux coccidies nouvelles, parasites de l'épinoche et de la sardine (Ann. de micrographie, 1890, II, p. 475–483).

10. *A. Schneider*, Monographie der Nematoden, 1866.

11. *V. Linstow*, Helminthologische Untersuchungen (Jahreshefte d. Ver. J. Vaterländische Naturkunde in Wurttemberg, 1879, p. 323). Fig. 7.

12. *V. Linstow*, Bemerkungen uber Mermis (Arch. f. Mikr. Anat. 1889, p. 392–396, Taf. XXII, 2–8).

13. *V. Linstow*, Weitere Beobachtungen an Gordius tolanus und Mermis (Arch. cit., 1891, p. 239–249).

14. *V. Siebold*, Stettiner Entomol. Zeitung, 1848, p. 299.

15. *Kraemer*, Merinthoidium mucronatum (Münchener illust. Zeitung, III, 1855, p. 291, Tf. XI.

## Figures and Tables

**Fig. 1. Fig. 2. Fig. 3. Fig. 4. Fig. 5. Fig. 6. Fig. 7. f1:**
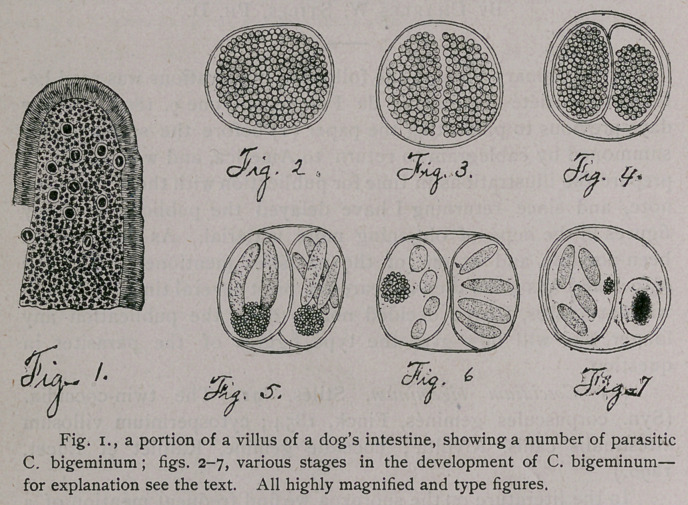


**Fig. 8. f2:**
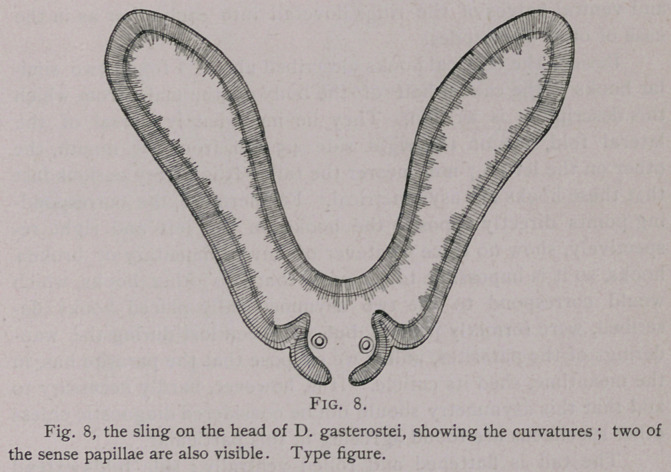


**Fig. 9. Fig. 10. f3:**
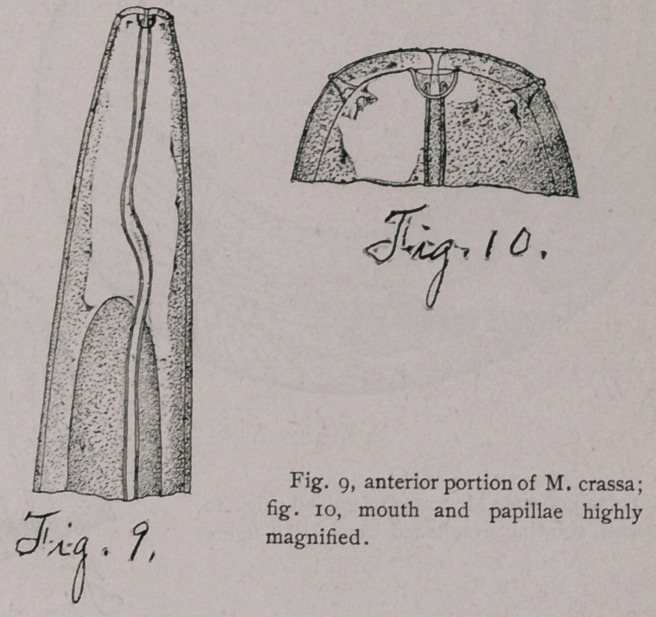


**Fig. 11. Fig. 12. f4:**